# Habitat Associations of Bats in an Agricultural Landscape: Linear Features Versus Open Habitats

**DOI:** 10.3390/ani10101856

**Published:** 2020-10-12

**Authors:** Domhnall Finch, Henry Schofield, Fiona Mathews

**Affiliations:** 1 School of Life Sciences, University of Sussex, Falmer, Brighton BN1 9QG, UK; d.finch@sussex.ac.uk; 2 Vincent Wildlife Trust, Bronsil Courtyard, Eastnor, Ledbury, Herefordshire HR8 1EP, UK; henryschofield@vwt.org.uk

**Keywords:** agriculture, agri-environmental scheme, bats, connectivity, fragmentation, habitat degradation, hedgerow management, *Pipistrellus pipistrellus*, *Pipistrellus pygmaeus*, *Rhinolophus ferrumequinum*

## Abstract

**Simple Summary:**

Understanding how species interact with agricultural landscapes is essential for future conservation efforts. Using a large-scale citizen science project, we examined the influence linear features have on bat activity compared to the centre of agricultural fields and detailed the effect of different types of linear features (intensively managed hedgerows, sympathetically managed hedgerows and treelines). Our results showed that all 10 bat species groups identified during surveys occurred both in the centre of fields as well as along linear features. Out of the five species groups analysed further, only *Nyctalus noctula* had no preference for linear features, compared to the centre of agricultural fields; however, 29% of *Rhinolophus ferrumequinum* calls were recorded in the centre of fields. More species were active near treelines compared to other linear feature types. Our results highlight the importance of linear feature management to bat conservation, but also the need to consider field centres during survey design and Environmental Impact Assessments

**Abstract:**

1. Bats are important components of agricultural ecosystems. However, little is known about the extent to which bats use linear features when foraging and commuting in agricultural settings, when compared to the interior of fields. 2. As part of a large-scale citizen science project, bat detectors were placed in the centre of agricultural fields (arable and pasture) and along linear features (intensively managed hedgerows, sympathetically managed hedgerows and treelines). 3. Our results show that all 10 of the bat species groups identified were found both along linear features and in the middle of agricultural fields. Of the five species groups analysed further, all had significantly more bat activity along linear features compared to the middle of fields, except for *Nyctalus noctula*. However, our results showed that 29% of calls from *Rhinolophus ferrumequinum* were recorded in the middle of agricultural fields, compared to only 10% for *Pipistrellus pipistrellus*. Bat activity was more likely to be associated with treelines compared to other linear feature types. 4. Our results highlight the importance of linear feature management to bat conservation, but also the need to consider field centres during survey design and Environmental Impact Assessments.

## 1. Introduction

Agricultural intensification is considered one of the most important drivers of global declines in biodiversity, through habitat loss, transformation and fragmentation [1]. In the past, agricultural landscapes were structurally heterogeneous, consisting of a myriad of agricultural, and semi-natural habitats in close proximity, supporting high biodiversity e.g., [2]. However, over the last 100 years, agricultural land has become more homogeneous, with increased land parcel sizes having high negative impacts on wildlife [3,4]. To facilitate this increase in parcel size, many historical linear features have been removed altogether from Europe’s landscapes, in some cases as much as 71% [5].

Treelines and hedgerows play a crucial role for biodiversity by providing structural heterogeneity, foraging grounds, breeding habitat and functional connectivity in the wider landscape for numerous taxa from small mammals and bats [6,7], to birds [8] and invertebrates [9]. Additionally, these linear features provide ecological services such as reducing soil erosion, increasing water retention and reducing pest incidences, as well as providing food and shelter for farm livestock [10]. Despite the historic loss, unfavourable management and neglect of hedgerows and treelines, their ecological importance is recognised and they are a priority habitat across Europe; financial incentives are provided for their conservation and management through Agri-Environmental Schemes (AES) [11].

Many European bat species are highly associated with linear features, e.g., [12,13]. These features have been shown to increase functional connectivity and permeability into the environment at a landscape scale, thus reducing the ‘barrier effect’ caused by other features such as streetlights and roads [14,15,16]. However, while there is some research about how distance to the nearest linear feature can influence bat activity, e.g., [7], the potential importance of open habitats are quite often overlooked, compared to linear features, for species other than *Nyctalus* and *Eptesicus* bats, e.g., [17], without quantifying relative amounts of activity in each habitat type. Recent research illustrated the importance of cattle-grazed fields, with both *Rhinolophus ferrumequinum* and *Pipistrellus pipistrellus* showing significantly more activity there compared to un-grazed fields [18]. These results highlight the relative importance of certain open agricultural habitats for bats, regardless of the amount of edge habitat or natural vegetation surrounding the site. However, at a larger scale, Heim et al. [19] showed that the amount of woodland and tree groves surrounding grassland sites is an important indicator for species activity and richness.

Using static bat detectors, we intend to examine how British bat species utilise landscape features within an agricultural setting. Here, we predict that both occurrence and relative activity of bat communities will be higher along linear features compared to open agricultural fields (both arable and pasture). In addition, we tested whether bat activity differed between three different linear feature types: sympathetically managed hedgerows, intensively managed hedgerows and treelines. We hypothesised that those linear features that are less managed will have higher activity along them compared to those that are more intensively managed. We also examine how historical linear features might influence the abundance of species records found in present day open agricultural fields using the ROAM database (http://digimap.edina.ac.uk/roam/historic). Overall, we aimed to add to the current literature of how bat species interact with agricultural landscapes.

## 2. Materials and Methods 

### 2.1. Site Selection

Sites were selected at four *R. ferrumequinum* roost sustenance zones (zones: area of land within 4 km of a roost) in Devon (England), because *R. ferrumequinum* is the rarest species in the landscape compared to the other species examined within this study, to maximising the probability of detecting this species. *R. ferrumequinum* roost size differed between the four zones: Zone 1 = 1187, Zone 2 =816, Zone 3 = 435 and Zone 4 = 260. First, at two zones, detectors were placed in a paired design (*n* = 24) to examine the extent to which bat communities use open, non-organic, agricultural fields (arable (*n* = 11) or pasture (*n* = 13) field) versus the linear features in the landscape surrounding them. None of the pasture fields had livestock grazing on them, except one, which had sheep. The main crop in the arable fields were barley and maize. Sites could only be selected at these two zones (Zone 1 and 2), instead of all four, due to restrictions in obtaining landowner permission. Second, to determine how bats utilised different linear feature types within an agricultural setting, 106 sites were selected across the four zones (these were placed around agricultural fields in the four zones, including those sites used in the paired experiment above); these sites included intensively managed hedgerows (*n* = 17), sympathetically managed hedgerows (*n* = 45) and treelines (*n* = 44). These were chosen based on the relative approximate habitat availability of these features within the landscape and the access for the volunteers to them. Linear features were defined as follows: intensively managed hedgerows are those hedgerows typically cut annually and that have a median height < 2m, sympathetically managed hedgerows are those with a median height > 2m that have not been cut in the previous year and treelines are defined as those sympathetically managed hedgerows > 6 m that have trees along the length of the feature, e.g., [16,20]. An example of both intensively managed and unmanaged hedgerows can be found in the Supplementary Material (Figure S1). Maps of the locations of each of the four zones, the habitat within them and each of the survey sites can be found in the Supplementary Material (Figures S2–S6).

### 2.2. Acoustic Surveys

Bat activity data (total number of passes per night) were collected between the 26th July and the 11th September 2016, as part of a large citizen science project (Devon Greater Horseshoe Bat Project). Volunteers were asked to place full spectrum bat detectors (SM2 and SM2+ detectors with an SMX-U1 and SMX-US ultrasonic microphone, Wildlife Acoustics, Maynard, MA, USA) out at the specific locations previously identified as being useful for the study, the 24 paired locations and the 106 locations along linear features, as described above. All detectors were pre-set to the manufacturer’s specifications before being placed out by the volunteers. Details of the acoustic detector settings are provided in Supplementary Material Table S1. Microphones were placed at a height of at least 1 m off the ground and were orientated horizontally. The detectors were set to record from 30 min before sunset to 30 min after sunrise, for a period of up to seven nights.

All bat passes were analysed using Kaleidoscope Pro software (version 3.1.1; Bats of Europe classifier version 3.0.0; Wildlife Acoustics, Maynard, MA, USA) and were verified manually on the basis of call frequency, shape and repetition rate to either species or genus level. A bat pass was defined as one or more echolocation call(s) within one second of each other, as done in previous studies [21,22]. Detailed characteristics of the ecological traits of all species examined in this study, including their call structure and their foraging strategies, can be found in Russ [23] and Dietz and Kiefer [24]. Supporting data for this study have been deposited on Figshare digital repository (10.6084/m9.figshare.12379922).

### 2.3. Statistical Analysis

Statistical analyses were undertaken using R version 3.3.0 [25]. To investigate the relationship between bat activity and agricultural landscape, generalised linear mixed models with a negative binomial distribution were built for total bat activity and for the activity of four species for which sufficient data was available (*Nyctalus noctula, P. pipistrellus, Pipistrellus pygmaeus* and *R. ferrumequinum*) using the ‘lme4′ package [26]. Three different models were created.

Model one tested for differences in bat activity according to habitat type, linear features versus the centre of agricultural fields. The activity (number of passes per night) of each of the five species groups was used as the response variable and the model included habitat type as the sole fixed factor and zone (two zones; data could only be collected at two out of the four zones surveyed due to land owner permissions), site (unique paired field ID) and individual detector identity as random factors to account for the potential non-independence of data gathered on consecutive nights and the surrounding landscape heterogeneity. Detector identity was nested within site, to account for the paired structure of the study. Excluding the one field containing sheep did not qualitatively change the results so it was kept in the analysis.

Model two explored whether total bat activity per night (response variable) depended on (i) distance to linear feature, (ii) field type (arable or pasture field) and (iii) whether historical linear features were present or not in the past (present at 17 out of the 24 sites; ROAM database), included as fixed effects in the model. Zones (two zones) was included as a random effect. This model was a subset of data recorded by those detectors placed in the middle of the agricultural fields only.

Model three tested whether bat activity per night differed between the types of linear features, included as a fixed effect, along with individual detector identity as a random factor. Zone (four zones) was used as a fixed factor for *R. ferrumequinum* because roost size differed between zones. Conversely, zone was used as a random factor for total bat activity and each of the other three species to account for any differences in landscape heterogeneity, which may occur between the four zones, which could have influenced their recorded activity levels. For significant fixed effects, we used multi-comparison Tukey adjusted post-hoc tests with the ‘lsmeans’ package [27] to test for pairwise differences between levels of the relevant factors (linear feature: three levels; Zones: four levels). For all models, a Bonferroni correction was applied to account for multiple testing for the four different species studied here, with a critical *p*-value set to 0.013. However, as Bonferroni corrections yields conservative estimates, the effects with *p*-values close to the critical value shown to enable readers to draw their own conclusions.

In addition, models one and three were repeated using generalised linear mixed models with a binary distribution (presence/absence as the response variable) for each of the four species studied (*N. noctula, P. pipistrellus, P. pygmaeus* and *R. ferrumequinum*). This was done to control for between-individual heterogeneity in recorded activity levels, ensuring that any results from the bat activity data were not biased by a few very active individuals.

## 3. Results

Nine species of bat were identified: *Barbastella barbastellus, Eptesicus serotinus, N. noctula, Pipistrellus nathusii, P. pipistrellus, P. pygmaeus, Plecotus auritus, Rhinolophus hipposideros, R. ferrumequinum*; and the genus Myotis. All species recorded occurred both in the middle of fields and along linear features. Sufficient data were available to examine relative activity for all species combined (thereafter referred to as total species) and four individual species out of the nine identified (*R. ferrumequinum, P. pipistrellus, P. pygmaeus* and *N. noctula*).

Total activity of all species recorded was higher along linear features compared to the middle of agricultural fields (Odds Ratio (OR): 4.11, 95% Confidence Intervals (CI): 2.74–6.16). The same difference was observed for three of the species taken individually (Table 1): *R. ferrumequinum* (OR: 3.51, CI: 1.90–6.47), *P. pipistrellus* (OR: 7.14, CI: 4.35–11.42) and *P. pygmaeus* (OR: 7.0, CI: 3.49–14.01)). Conversely, *N. noctula* was the only species to show no difference (OR: 1.10, CI: 0.69–1.74).

Using the subset of data restricted to records in the middle of agricultural fields, we found that distance to linear feature (median: 76 m, range: 30m–147 m; χ² = 0.003, *p* = 0.806), field type (χ² = 0.327, *p* = 0.503) and the presence of historical ROAM linear features (χ² = −0.464, *p* = 0.384) did not significantly influence total bat activity.

**Figure 1:**
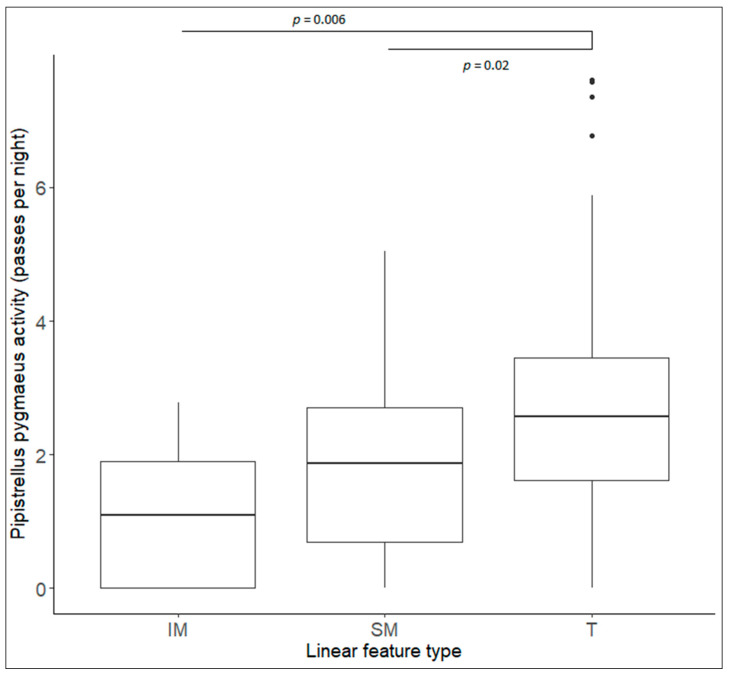
*Pipistrellus pygmaeus* activity (logged) along intensively managed hedgerows (IM), sympathetically managed hedgerows (SM) and treelines (T). Boxplots showing median activity with the black dots representing outliers.

Total bat activity did not significantly differ between linear feature types (χ² = 3.11, *p* = 0.21). Similar results were obtained for both *P. pipistrellus* (χ² = 2.71, *p* = 0.26) and *N. noctula (*χ² = 2.76, *p* = 0.25). However, the activity of *R. ferrumequinum* (χ² = 6.10, *p* = 0.047) and *P. pygmaeus* (χ² = 16.19, *p* < 0.001) significantly differed between linear feature types. Based on a post-hoc test, bat activity was significantly lower in intensively managed hedgerows compared to treelines for *R. ferrumequinum* (χ² = −0.91, *p* = 0.04) and *P. pygmaeus* (χ² = −1.38, *p* = 0.006; Figure 1) activity when compared to treelines. Similarly, a significantly higher activity was recorded along treelines when compared to sympathetically managed hedgerow for *P. pygmaeus* (χ² = 0.81, *p* = 0.02). There was no significant difference in *R. ferrumequinum* activity between sympathetically managed hedgerows and treelines (χ² = 0.05, *p* = 0.98), and activity was also similar on sympathetically managed and intensively managed hedgerows (χ² = 0.86, *p* = 0.06; Table 2). Activity of *R. ferrumequinum* significantly differed between zones (χ² = 10.63, *p* = 0.014), with significantly more activity recorded at only Zone 1 (χ² = −1.32, *p* = 0.01) and Zone 2 (χ² = 1.41, *p* < 0.001) compared to Zone 4.

Similar results were observed when using presence/absence data compared to activity data for each of the four species studied. The only key difference was that the presence of *R. ferrumequinum* was significantly higher along sympathetically managed hedgerows (χ² = 1.88, *p* = 0.001) compared to intensively managed hedgerows. Full details on all of the outputs from each model using presence/absence data can be found in the Supplementary Material.

## 4. Discussion

This research demonstrates the important influence that linear features have on bat activity in an agricultural landscape, illustrating the need for their protection and appropriate management. Along with total bat activity, illustrating a general pattern, three of the four species analysed showed significantly higher bat activity at linear features compared with the middle of fields. Additionally, the frequency at which bats were recorded in the middle of fields differed between species. Our results are in line with previous studies, which report lower bat activity for open habitats compared to linear features, e.g., [7,11,12,17]. The attraction of bats to linear features can be largely explained by higher food availability, protection from predators and wind [13,28] and the use of linear features for navigation [29].

Out of those species with significantly higher activity close to linear features, we identify that nevertheless, almost a third, i.e., 29%, of *R. ferrumequinum* activity is recorded in the centre of agricultural fields. In this study, *R. ferrumequinum* were found along every linear feature surveyed but were also found in all but three of the paired agricultural fields. Such findings are unusual for species that tend to be heavily associated with linear features or woodlands, e.g., [30]. This highlights the importance of such areas when designing acoustic surveys and the need to include ‘sub-optimal’ habitat into Environmental Impact Assessments, to get an accurate understanding of how bats are utilising the landscape throughout the year.

Using the ROAM database, we assessed whether the relative activity of bats in the middle of fields could be due to a historical legacy of hedgerows being present in those locations in the past, acting as old commuting routes and foraging grounds for the bats. However, our results demonstrated that this did not seem to be the case, no difference in bat activity (either in total or specifically for *R. ferrumequinum*) recorded between fields that did and did not have previous linear features in their centres since the earliest ROAM records for these locations in the 1930s.

Overall, our findings show that two species out of four were significantly more associated with treelines and unmanaged hedgerows compared to intensively managed hedgerows. This reiterates the results from Wickramasinghe et al. [31], Brandt et al. [32] and Froidevaux et al. [20], who found that increased bat activity and foraging potential occurred along linear features on agricultural land that had trees and taller shrubs present. This is especially true for bat species associated with woodlands and woodland-edge habitat [33,34,35]. Boughey et al. [11] found that unlike height, hedgerow width did not influence bat activity, but the length of individual hedgerows and the total length at which they occur in the surrounding landscape are important for bat activity [17]. It is not only bats whose activity is significantly associated with well-developed treelines and taller hedgerows. The latter have been shown to be associated with increased floral diversity, as well as moth species richness and bird abundance/density [36,37,38,39]. Similar to their effect on bats, they can act as food stores, shelter belts (creating microclimates) and breeding/roosting locations for other species [8,37,40]. However, this increase in diversity along linear features critically depends on their management, with those that are cut every three years showing some of the highest benefit for biodiversity [20,37]. Reducing cutting frequency from every year to every three years was shown to result in 2.1 times more flowers and a 3.4 times greater berry mass over five years [37]. These associations and benefits may be due to the structural changes in linear features due to their succession from sympathetically managed hedgerows to treelines [36].

As discussed above, linear features are critically important for a wide variety of species, for feeding, roosting and movement of wildlife, particularly in a fragmented landscape, yet they are under threat. From a UK perspective, the total length of linear features has decreased by approximately 23% over 16 years [41] and the number of trees present within them has also decreased by 6.6% over a 20 year period [42]. Boughey et al. [11] speculated that this is due to management regimes, illustrating the point that new trees in hedgerows add cost to mechanical trimming and decrease crop production through shading. However, as our results indicate, increased linear feature height was associated with greater bat activity, having the potential to act as critical corridors and foraging areas for the long-term survival of populations. Such corridors provide even more important resources during periods of lactation, as females tend to travel shorter distances to forage; increased travel time to foraging grounds can negatively impact juvenile growth and survivorship [43,44]. Appropriately managing, retaining and rebuilding the countryside’s network of linear features, particularly those of higher quality (sympathetically managed hedgerows and treelines), through result-based financial incentives from AES (or otherwise), is vital to the conservation of bats and the many other species using them [20,39]. Appropriate ecological assessments and considerations of cumulative impacts at a landscape scale need to be given when examining the effects of agricultural practices on biodiversity.

## 5. Conclusions

In conclusion, we demonstrate the importance of linear features for the activity of bat communities in agricultural landscape, when compared to the centre of agricultural fields. However, the rate of activity along these features differs between species, with no significant difference in *P. pipistrellus* and *N. noctula* activity being observed between the different linear feature types. Conversely, *R. ferrumequinum* and *P. pygmaeus* showed higher activity along those linear features that are taller and less intensively managed (unmanaged hedgerows and treelines). Nevertheless, our results indicate that high levels of *R. ferrumequinum* activity, i.e., 29%, occurred in the centre of agricultural fields. These results stress the need for appropriate survey designs to include all aspects of the landscape, including habitats that are perceived as ‘sub-optimal’, particularly for rarer bat species during Environmental Impact Assessments.

## Figures and Tables

**Table 1:** Bat species activity recorded along linear features compared to in the middle of agricultural fields.

Species or Genus.	Total No. of Passes Recorded		Mean No. of Passes per Night, SD and the Percentage			
	Field	Linear Feature	Field		Linear Feature	
			Mean	SD	Mean	SD
*Rhinolophus ferrumequinum*	225	396	1.6	3.5	3.9	4.4
*Pipistrellus pipistrellus*	482	3214	3.4	5.4	31.5	54.8
*Pipistrellus pygmaeus*	244	1036	1.7	3.9	10.2	19.3
*Nyctalus noctula*	536	419	3.8	8.3	4.1	9.2
Total species	1796	5679	12.8	13.6	55.7	67.9

**Table 2:** A comparison of bat species activity between the three different types of linear features: intensively managed hedgerows (IM), sympathetically managed hedgerows (SM) and treelines (T).

Species or Genus.	Total No. of Passes Recorded			Mean No. of Passes per Night, SD and the Percentage								
	IM	SM	T	IM			SM			T		
				Mean	SD	%	Mean	SD	%	Mean	SD	%
*Rhinolophus ferrumequinum*	618	1174	3268	5.8	10.4	15	5.0	8.3	14	26.8	80.8	71
*Pipistrellus pipistrellus*	3074	13,304	7923	28.7	47.6	19	56.4	137.6	38	64.9	103.4	43
*Pipistrellus pygmaeus*	443	2511	9597	4.1	11.9	5	10.6	21.5	11	78.7	297.1	84
*Nyctalus noctula*	293	931	421	2.7	8.7	27	3.9	8.8	38	3.5	6.2	35
Total species	5724	20,846	26,370	53.5	66.6	15	88.3	55.4	25	216.1	499.2	60

## Supplementary Materials

The following are available online at https://www.mdpi.com/2076-2615/10/10/1856/s1, Figure S1: Detector settings used for both the SMX-U1 and SMX-US microphones used in conjunction with SM2 and SM2 bat+ detectors (Wildlife Acoustics, USA) during the acoustic bat surveys., Figure S1: Image depicting a typical intensively managed hedgerow (left) and an unmanaged hedgerow (right), Figure S2: Map showing the location of the four greater horseshoe bat roosts used to define the study zones in Devon, England., Figure S3: Map illustrating the general mosaic of landscape features surrounding Zone 1 and the locations of the acoustic static bat detectors (linear feature or agricultural land). Paired detectors (centre of agricultural fields vs. linear features) could not always be sited within the same field and therefore had to be placed in adjacent fields due to authorisation issues. Map for illustrative purposes only, Figure S4: Map illustrating the general mosaic of landscape features surrounding Zone 2 and the locations of the acoustic static bat detectors (linear feature or agricultural land). Paired detectors (centre of agricultural fields vs. linear features) could not always be sited within the same field and therefore had to be placed in adjacent fields due to authorisation issues. Map for illustrative purposes only, Figure S5: Map illustrating the general mosaic of landscape features surrounding Zone 3 and the locations of the acoustic static bat detectors (linear feature). Map for illustrative purposes only, Figure S6: Map illustrating the general mosaic of landscape features surrounding Zone 4 and the locations of the acoustic static bat detectors (linear feature). Map for illustrative purposes only.

## References

[1] Foley J.A., Ramankutty N., Brauman K.A., Cassidy E.S., Gerber J.S., Johnston M., Mueller N.D., O’Connell C., Ray D.K., West P.C. (2011). Solutions for a cultivated planet. Nature.

[2] Weibull A.C., Bengtsson J., Nohlgren E. (2000). Diversity of butterflies in the agricultural landscape: The role of farming system and landscape heterogeneity. Ecography.

[3] Robinson R.A., Sutherland W.J. (2002). Post-war changes in arable farming and biodiversity in Great Britain. J. Appl. Ecol..

[4] Benton T.G., Vickery J.A., Wilson J.D. (2003). Farmland biodiversity: Is habitat heterogeneity the key?. Trends Ecol. Evol..

[5] Sklenicka P., Molnarova K., Brabec E., Kumble P., Pittnerova B., Pixova K., Salek M. (2009). Remnants of medieval field patterns in the Czech Republic: Analysis of driving forces behind their disappearance with special attention to the role of hedgerows. Agric. Ecosyst. Environ..

[6] Gelling M., Macdonald D.W., Mathews F. (2007). Are hedgerows the route to increased farmland small mammal density? Use of hedgerows in British pastoral habitats. Landsc. Ecol..

[7] Kelm D.H., Lenski J., Kelm V., Toelch U., Dziock F. (2014). Seasonal bat activity in relation to distance to hedgerows in an agricultural landscape in central Europe and implications for wind energy development. Acta Chiropt..

[8] Hinsley S.A., Bellamy P.E. (2000). The influence of hedge structure, management and landscape context on the value of hedgerows to birds: A review. J. Environ. Manag..

[9] Dover J., Sparks T. (2000). A review of the ecology of butterflies in British hedgerows. J. Environ. Manag..

[10] Baudry J., Bunce R., Burel F. (2000). Hedgerows: An international perspective on their origin, function and management. J. Environ. Manag..

[11] Boughey K.L., Lake I.R., Haysom K.A., Dolman P.M. (2011). Improving the biodiversity benefits of hedgerows: How physical characteristics and the proximity of foraging habitat affect the use of linear features by bats. Biol. Conserv..

[12] Walsh A.L., Harris S. (1996). Foraging habitat preferences of vespertilionid bats in Britain. J. Appl. Ecol..

[13] Verboom B., Spoelstra K. (1999). Effects of food abundance and wind on the use of tree lines by an insectivorous bat, *Pipistrellus pipistrellus*. Can. J. Zool..

[14] Stone E., Jones G., Harris S. (2009). Street lighting disturbs commuting bats. Curr. Biol..

[15] Berthinussen A., Altringham J. (2012). The effect of a major road on bat activity and diversity. J. Appl. Ecol..

[16] Finch D., Corbacho D.P., Schofield H., Davison S., Wright P.G., Broughton R.K., Mathews F. (2020). Modelling the functional connectivity of landscapes for greater horseshoe bats *Rhinolophus ferrumequinum* at a local scale. Landsc. Ecol..

[17] Verboom B., Huitema H. (1997). The importance of linear landscape elements for the pipistrellePipistrellus pipistrellus and the serotine batEptesicus serotinus. Landsc. Ecol..

[18] Ancillotto L., Ariano A., Nardone V., Budinski I., Rydell J., Russo D. (2017). Effects of free-ranging cattle and landscape complexity on bat foraging: Implications for bat conservation and livestock management. Agric. Ecosyst. Environ..

[19] Heim O., Treitler J.T., Tschapka M., Knörnschild M., Jung K. (2015). The importance of landscape elements for bat activity and species richness in agricultural areas. PLoS ONE.

[20] Froidevaux J.S., Boughey K.L., Hawkins C.L., Broyles M., Jones G. (2019). Managing hedgerows for nocturnal wildlife: Do bats and their insect prey benefit from targeted agri-environment schemes?. J. Appl. Ecol..

[21] Fenton M.B. (1970). A technique for monitoring bat activity with results obtained from different environments in southern Ontario. Can. J. Zool..

[22] Walsh A., Harris S. (1996). Determinants of vespertilionid bat abundance in Britain: Geographic, land class and local habitat relationships (II). J. Appl. Ecol..

[23] Russ J. (2012). British Bat Calls: A Guide to Species Identification.

[24] Dietz C., Kiefer A. (2016). Bats of Britain and Europe.

[25] R Core Team (2016). R: A Language and Environment for Statistical Computing.

[26] Bates D., Mächler M., Bolker B., Walker S. (2015). Fitting Linear Mixed-Effects Models Using lme4. J. Stat. Softw..

[27] Lenth R. (2016). Least-Squares Means: The R Package lsmeans. R package versions 2.30-0. J. Stat. Softw..

[28] Downs N.C., Racey P.A. (2006). The use by bats of habitat features in mixed farmland in Scotland. Acta Chiropt..

[29] Schaub A., Schnitzler H.-U. (2007). Flight and echolocation behaviour of three vespertilionid bat species while commuting on flyways. J. Comp. Physiol. A.

[30] Billington G. (2008). Radio Tracking Study of Greater Horseshoe Bats at Dean Hall, Littledean, Cinderford.

[31] Wickramasinghe L.P., Harris S., Jones G., Vaughan N. (2003). Bat activity and species richness on organic and conventional farms: Impact of agricultural intensification. J. Appl. Ecol..

[32] Brandt G., Blows L., Linton D., Paling N., Prescott C. (2007). Habitat associations of British bat species on lowland farmland within the Upper Thames catchment area. Cent. Wildl. Assess. Conserv. E-J..

[33] Russ J., Montgomery W. (2002). Habitat associations of bats in Northern Ireland: Implications for conservation. Biol. Conserv..

[34] Nicholls B., Racey P. (2006). Habitat selection as a mechanism of resource partitioning in two cryptic bat species *Pipistrellus pipistrellus* and *Pipistrellus pygmaeus*. Ecography.

[35] Fuentes-Montemayor E., Goulson D., Cavin L., Wallace J.M., Park K.J. (2013). Fragmented woodlands in agricultural landscapes: The influence of woodland character and landscape context on bats and their insect prey. Agric. Ecosyst. Environ..

[36] MacDonald D., Johnson P. (1995). The relationship between bird distribution and the botanical and structural characteristics of hedges. J. Appl. Ecol..

[37] Staley J.T., Sparks T.H., Croxton P.J., Baldock K.C., Heard M.S., Hulmes S., Hulmes L., Peyton J., Amy S.R., Pywell R.F. (2012). Long-term effects of hedgerow management policies on resource provision for wildlife. Biol. Conserv..

[38] Merckx T., Marini L., Feber R.E., Macdonald D.W. (2012). Hedgerow trees and extended-width field margins enhance macro-moth diversity: Implications for management. J. Appl. Ecol..

[39] Froidevaux J.S., Broyles M., Jones G. (2019). Moth responses to sympathetic hedgerow management in temperate farmland. Agric. Ecosyst. Environ..

[40] Maudsley M. (2000). A review of the ecology and conservation of hedgerow invertebrates in Britain. J. Environ. Manag..

[41] Barr C., Gillespie M. (2000). Estimating hedgerow length and pattern characteristics in Great Britain using Countryside Survey data. J. Environ. Manag..

[42] Carey P., Wallis S., Chamberlain P., Cooper A., Emmett B., Maskell L., McCann T., Murphy J., Norton L., Reynolds B. (2008). Countryside Survey: UK results from 2007.

[43] Clark B.S., Leslie D.M., Carter T.S. (1993). Foraging activity of adult female Ozark big-eared bats (*Plecotus townsendii ingens*) in summer. J. Mammal..

[44] Kerth G., Melber M. (2009). Species-specific barrier effects of a motorway on the habitat use of two threatened forest-living bat species. Biol. Conserv..

