# Supplementary material for: Habitat Associations of Bats in an Agricultural Landscape: Linear Features Versus Open Habitats
Source: Animals (Basel). 2020 Oct 12;10(10):1856. doi: 10.3390/ani10101856 (PMC7650766; doi:10.3390/ani10101856)
Supplement: Supplementary file 1 [file animals-10-01856-s001.pdf]

Similar results were observed using binary (presence/absence) data compared with bat activity data. We demonstrate that Rhinolophus ferrumequinum (Odds ratio (OR): 6.86, 95% Confidence Intervals (CI): 2.68 – 17.61), Pipistrellus pipistrellus (OR: 4.28, CI: 1.96 – 9.328) and Pipistrellus pygmaeus (OR: 10.88, CI: 3.11 – 38.06) are more likely to be present along linear features compared to the centre of agricultural fields. Conversely, Nyctalus noctula was the only species to show no difference (OR: 0.91, CI: 0.42 – 1.96).

No significant difference in the presence of *P. pipistrellus* ($\chi^2 = 5.44$, $p = 0.066$) and *N. noctula* ($\chi^2 = 1.05$, $p = 0.593$) was observed between the three different linear feature types. However, a significant association with linear feature type was observed for both the presence of *R. ferrumequinum* ($\chi^2 = 12.58$, $p = 0.002$) and *P. pygmaeus* ($\chi^2 = 13.45$, $p = 0.001$). Based on a post-hoc test, more *R. ferrumequinum* were present along unmanaged hedgerows ($\chi^2 = 1.88$, $p = 0.003$) and treelines ($\chi^2 = 1.78$, $p = 0.006$) compared to intensively managed hedgerows. Similarly, more *P. pygmaeus* were present along unmanaged hedgerows ($\chi^2 = 1.77$, $p = 0.001$) and treelines ($\chi^2 = 1.73$, $p = 0.002$) compared to intensively managed hedgerows.

The presence of *R. ferrumequinum* significantly differed between zones ($\chi^2 = 11.01$, p-value = 0.012), with Zone 1 ($\chi^2 = 1.41$, $p = 0.05$) and Zone 2 ($\chi^2 = 2.20$, $p = 0.009$) showing higher presence records compared to Zone 4.

Table S1. Detector settings used for both the SMX-U1 and SMX-US microphones used in conjunction with SM2 and SM2 bat+ detectors (Wildlife Acoustics, USA) during the acoustic bat surveys.

Detector settings	SMX-U1	SMX-US
Sampling rate	192,000 kHz	192,000 kHz
Gain	12dB	48dB
High pass filter	4 kHz	4 kHz
Low pass filter	Off	Off
Trigger level	18 SNR	18 SNR
Trigger window	2.0 sec	2.0 sec

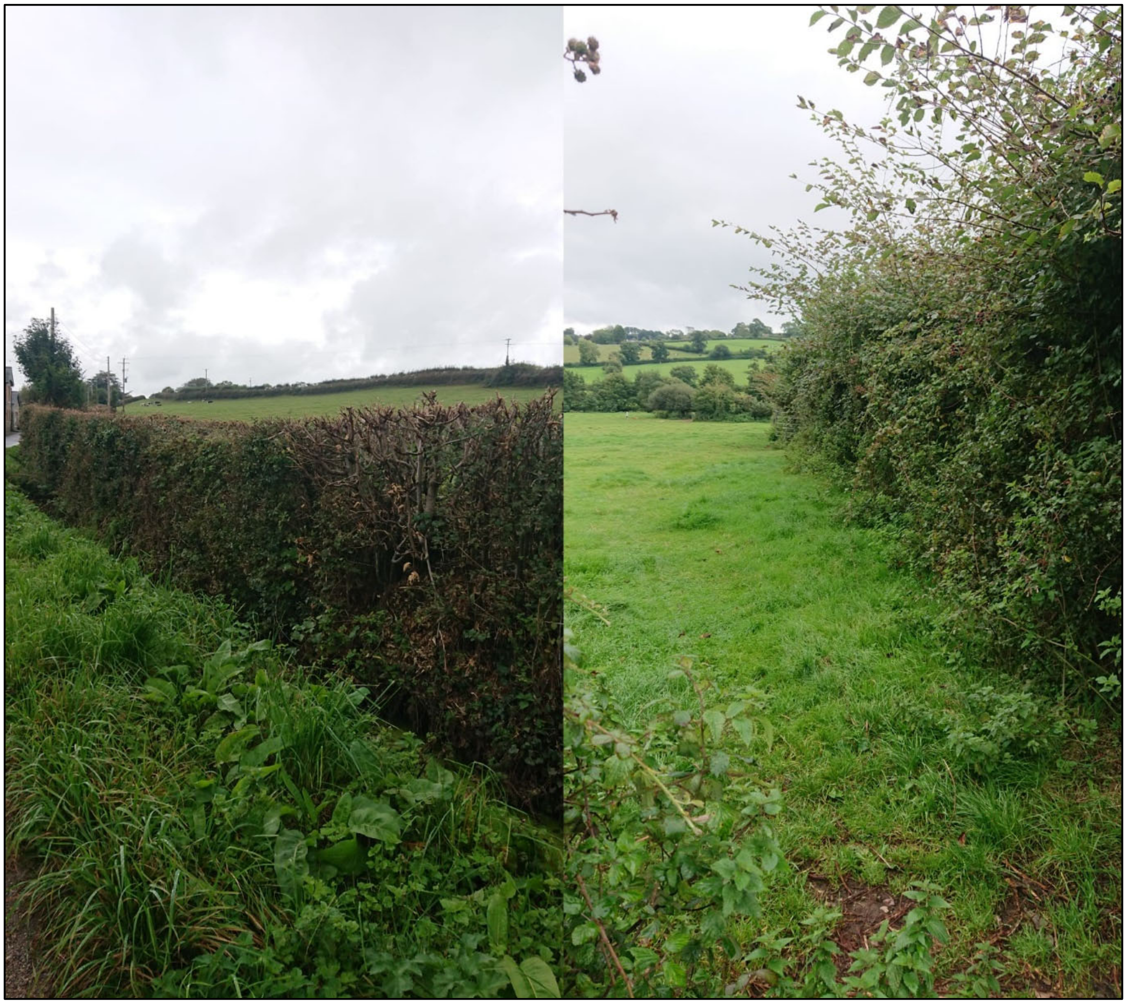

Figure S1. Image depicting a typical intensively managed hedgerow (left) and an unmanaged hedgerow (right).

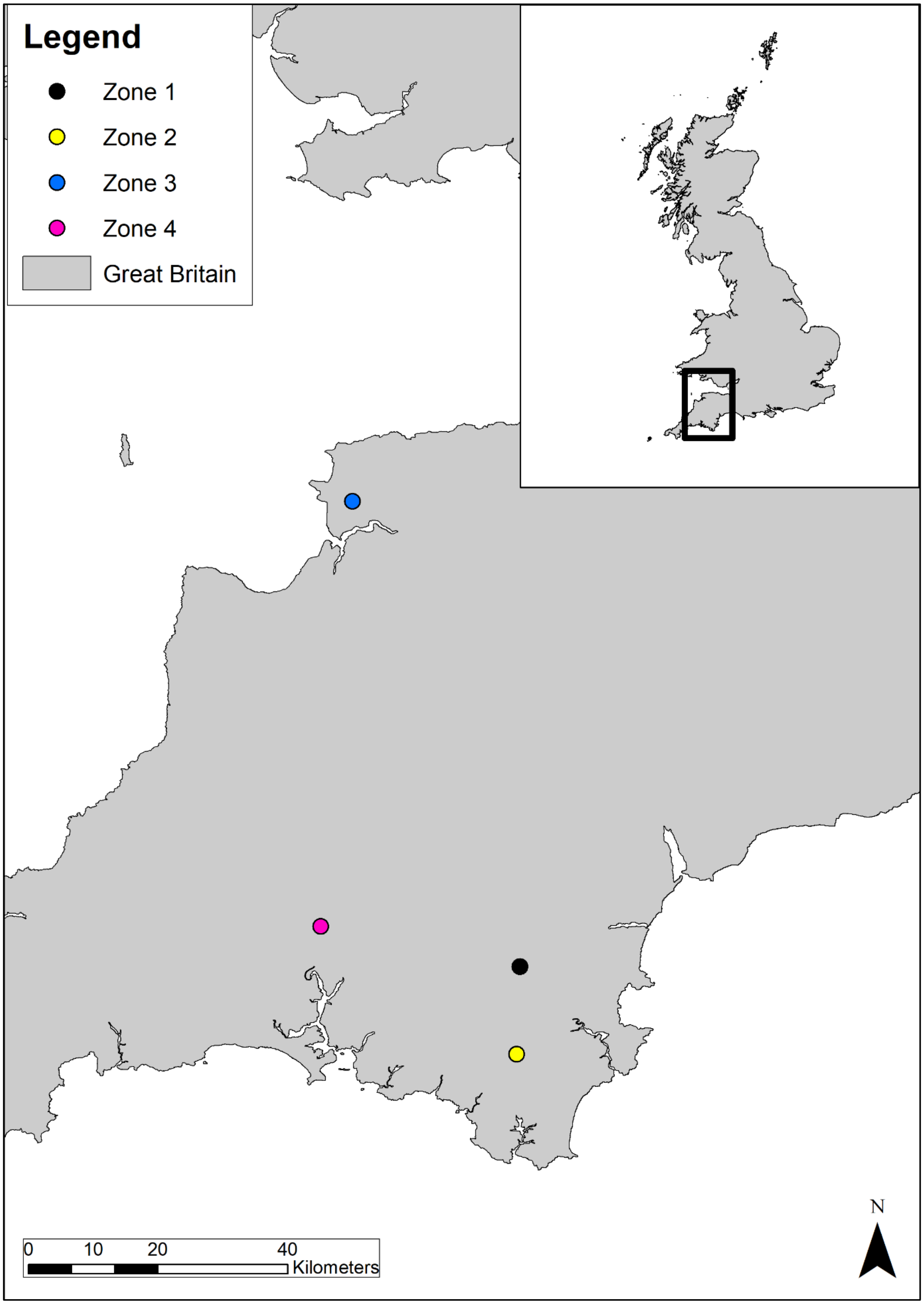

Figure S2. Map showing the location of the four greater horseshoe bat roosts used to define the study zones in Devon, England.

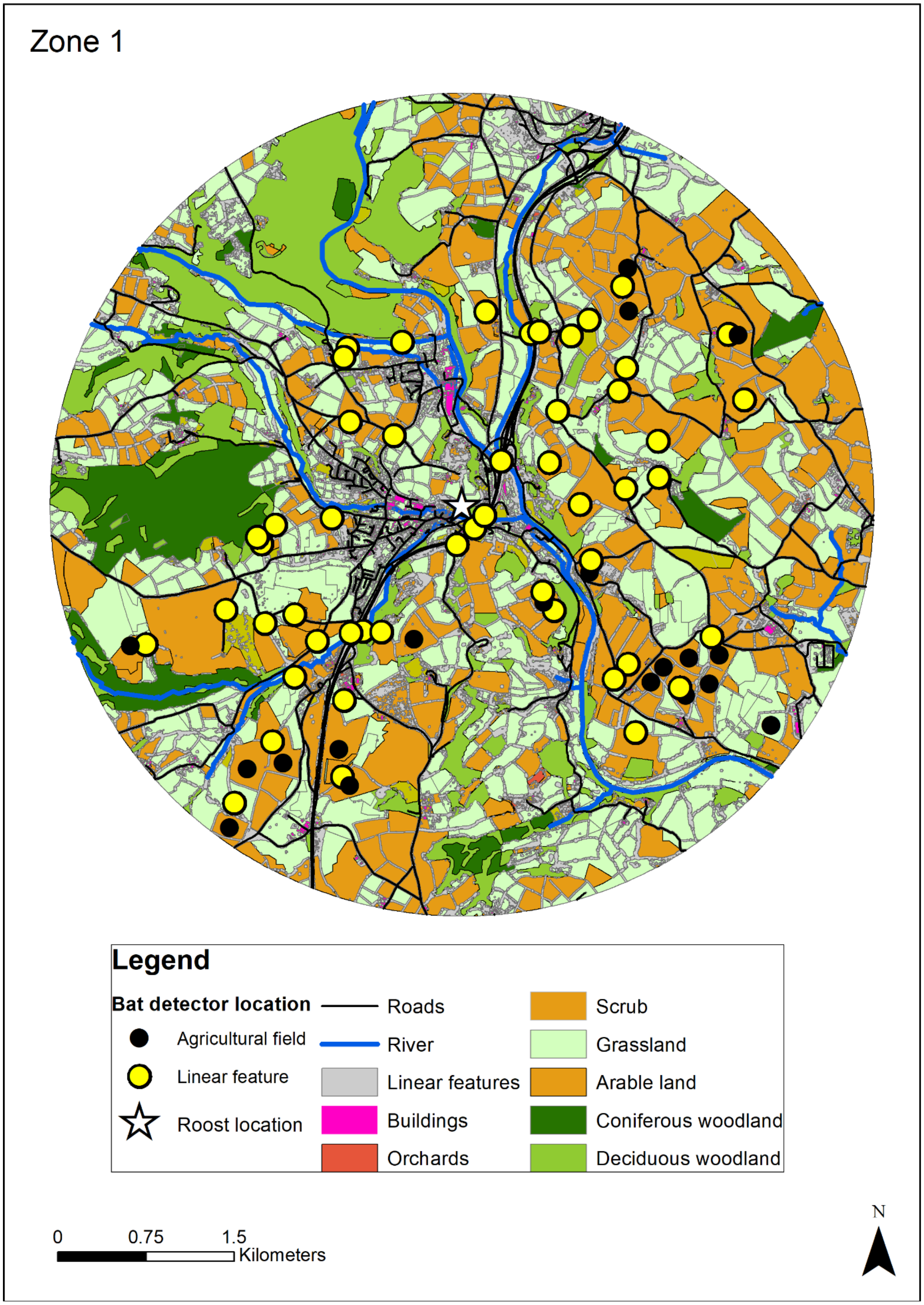

Figure S3. Map illustrating the general mosaic of landscape features surrounding Zone 1 and the locations of the acoustic static bat detectors (linear feature or agricultural land). Paired detectors (centre of agricultural fields vs. linear features) could not always be sited within the same field, and therefore, had to be placed in adjacent fields due to authorisation issues. Map for illustrative purposes only.

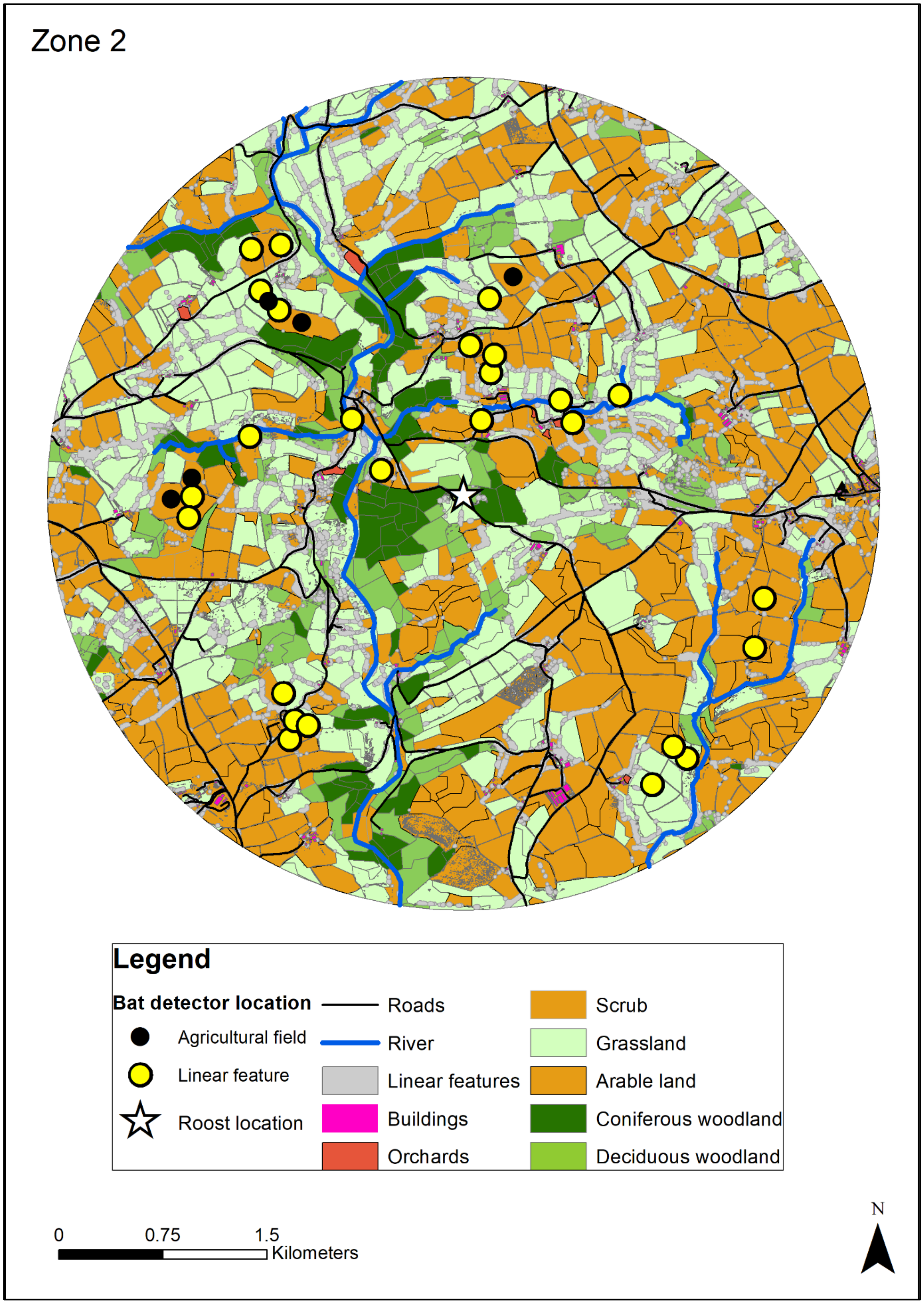

Figure S4. Map illustrating the general mosaic of landscape features surrounding Zone 2 and the locations of the acoustic static bat detectors (linear feature or agricultural land). Paired detectors (centre of agricultural fields vs. linear features) could not always be sited within the same field, and therefore, had to be placed in adjacent fields due to authorisation issues. Map for illustrative purposes only.

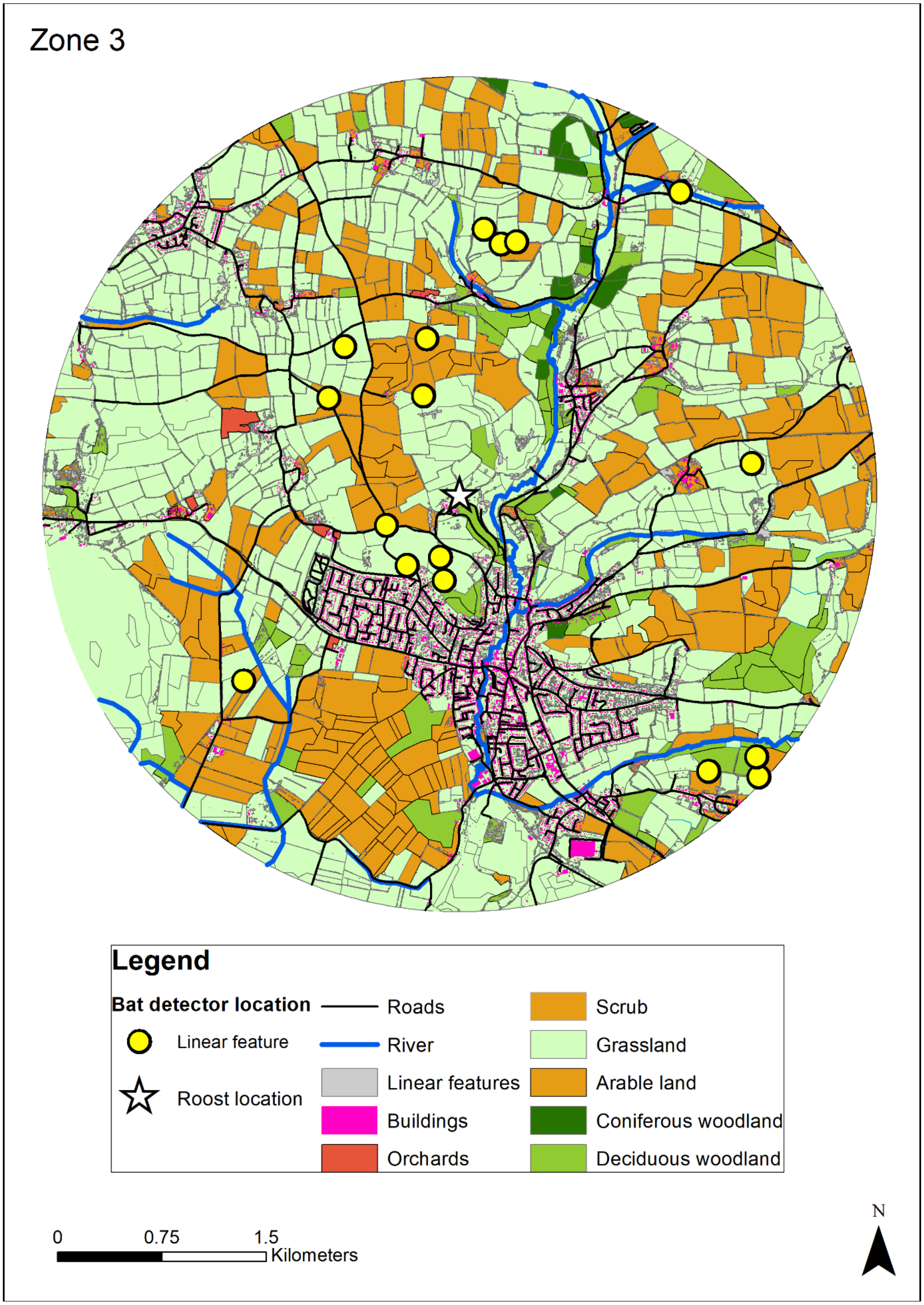

Figure S5. Map illustrating the general mosaic of landscape features surrounding Zone 3 and the locations of the acoustic static bat detectors (linear feature). Map for illustrative purposes only.

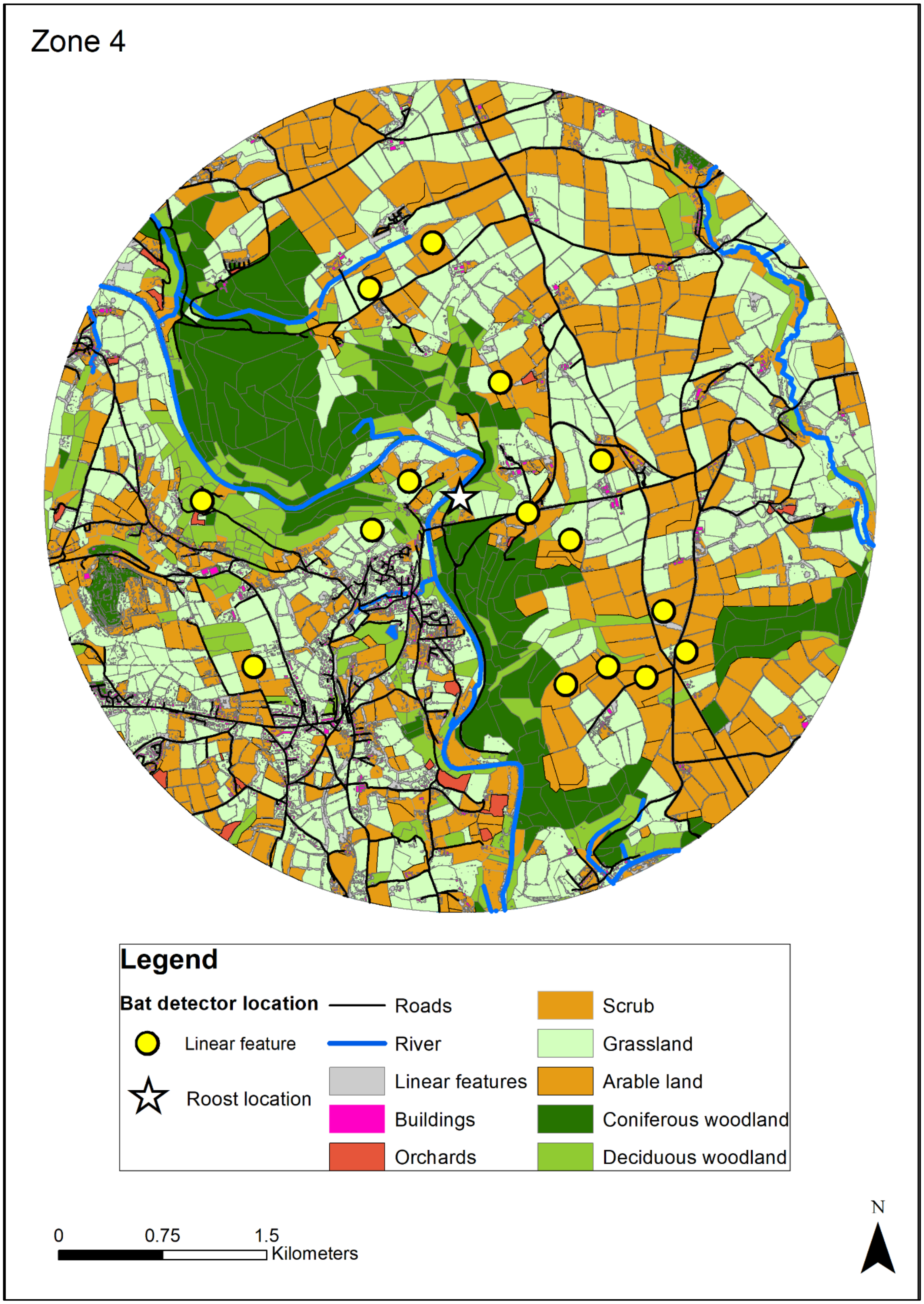

Figure S6. Map illustrating the general mosaic of landscape features surrounding Zone 4 and the locations of the acoustic static bat detectors (linear feature). Map for illustrative purposes only.
